# Metformin therapy associated with survival benefit in lung cancer patients with diabetes

**DOI:** 10.18632/oncotarget.8881

**Published:** 2016-04-20

**Authors:** Guoxing Wan, Xiongjie Yu, Ping Chen, Xianhe Wang, Dongfeng Pan, Xuanbin Wang, Linjun Li, Xiaojun Cai, Fengjun Cao

**Affiliations:** ^1^ Department of Oncology, Renmin Hospital, Hubei University of Medicine, Shiyan, Hubei, China

**Keywords:** lung cancer, metformin, survival, diabetes, meta-analysis

## Abstract

The purpose of this study is to summarize the currently available evidence regarding the concerned issue by performing a comprehensive meta-analysis. Relevant publications reporting the association of metformin use with survival of lung cancer patients with diabetes were electronically searched to identify eligible studies. The meta-analysis was performed with hazard ratios (HRs) and 95% confidence intervals (95% CIs) as effect measures for disease-free survival(DFS) and overall survival(OS) estimates. A total of 17 individual studies from 10 publications were included in the meta-analysis. Overall, the results revealed a significant association of metformin use with a better survival of lung cancer patients with diabetes(for DFS: HR = 0.65, 95%CI = 0.52-0.83; for OS: HR = 0.78, 95%CI = 0.64-0.93). The subgroup analyses showed similar association in Asian region(for DFS:HR = 0.69, 95%CI = 0.59-0.80; for OS: HR = 0.55, 95%CI = 0.46-0.67) but not in Western region. Such association was also presented in small cell lung cancer (for DFS: HR = 0.54, 95%CI = 0.38-0.77; for OS: HR = 0.52, 95%CI = 0.39-0.69) and in non-small cell lung cancer(for DFS: HR = 0.70, 95%CI = 0.51-0.96; for OS: HR = 0.75, 95%CI = 0.58-0.97). Analyses stratified by treatment strategy showed a reduction in the risk of cancer-related mortality in patients receiving chemotherapy(for DFS: HR = 0.71, 95%CI = 0.64-0.83; for OS: HR = 0.58, 95%CI = 0.47-0.71) but not in patients receiving chemoradiotherapy. The meta-analysis demonstrated that metformin use was significantly associated with a favorable survival outcome of lung cancer patients with diabetes.

## INTRODUCTION

According to the epidemiologic and clinical studies, approximately 8% to 18% of individuals survived with a diagnosis of cancer accompanying with diabetes, probably due to the shared risk factors of the diseases, such as greater body mass index, smoking, and the lack of exercise and their growing global prevalence [1, 2]. The contribution of diabetes mellitus to the increased cancer mortality in patients with lung cancer has been extensively suggested by evidences in recent years [3], for which a plausible explanation may be multifactorial, involving the effect of hyperinsulinemia, hyperglycemia, and inflammatory cytokines on promoting neoplastic proliferation, invasion of cancer cell, and thereby resulting in initiation and progression of the neoplasms [4, 5]. Conceivably, an increasing body of evidence supported the notion that several antidiabetic medications such as metformin, sulfonylureas and thiazolidinediones, may exhibit anticancer effects in various malignancies, including lung cancer [6–8].

Metformin as one of the most commonly prescribed oral antidiabetic agents for type 2 diabetes may acts by inhibiting hepatic gluconeogenesis [9], reducing insulin resistance [10] and decreasing inflammatory response [11], thus controlling circulating glucose and presenting the potential antitumor effect [5]. Although the exact mechanism was not fully understood as yet, some in vitro and in vivo studies have demonstrated with the increasing attention attached to the metformin-mediated therapy in lung cancer, that metformin may exert the antitumor activity through indirect (insulin-mediated) effects and direct effects on the inhibition of cancer cell proliferation, colony formation, migration, and invasion, and the induction of cell cycle arrest and apoptosis mediated by the ATM/LKB1/AMPK axis and mammalian target of rapamycin (mTOR)-signaling pathway [12–16].

Up till now, a number of epidemiological studies have investigated the prognostic significance of metformin use in lung cancer patients with diabetes, but obtained inconsistent results. Tan et al. reported that metformin may improve chemotherapy outcomes and survival for advanced non-small cell lung cancer (NSCLC) patients with type 2 diabetes [17]. Xu et al. also observed a beneficial survival of metformin use in small cell lung cancer(SCLC) patients with diabetes [18]. However, Ahmed et al. demonstrated that metformin use may have no effect on survival in patients with diabetes and locally-advanced, inoperable NSCLC treated with definitive chemoradiation [19]. The discrepancy may be due to the limited sample size or the inefficient power of an individual study with a special design in examining the complicated association to various extents. Although a previous meta-analysis has evaluated the effect of metformin use on the overall survival(OS) of lung cancer patients with diabetes [20], only limited studies were included and the effect of metformin use on the disease-free survival(DFS, including the progression- and recurrence-free survival) was not concluded. In an effort to bring more clarity to this issue, a meta-analysis of currently available relevant observational studies was carried out to systematically reassess the effect of metformin use on the survival outcome of lung cancer patients with diabetes.

## RESULTS

### Literature search

**Figure 1:**
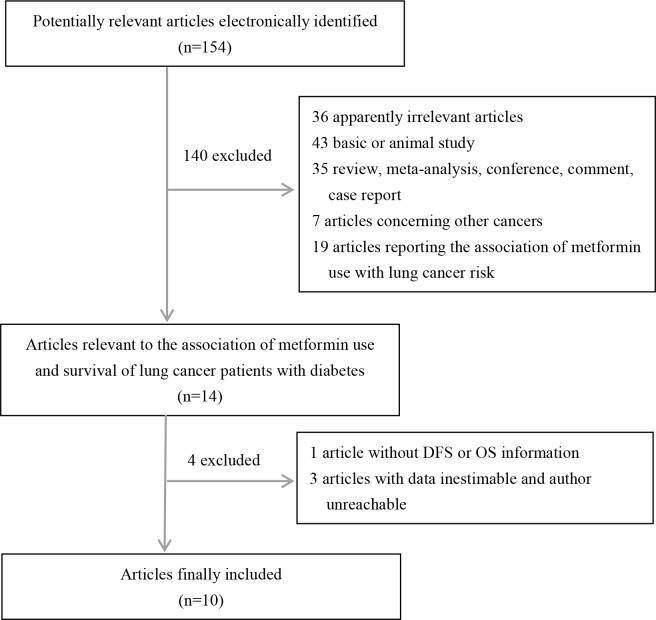
The participant flow diagram for the included studies

The participant flow diagram for the included studies is shown in Figure 1. Finally, a total of 10 publications evaluating the effect of metformin use on the survival outcome of lung cancer patients with diabetes were included in the meta-analysis. All the included studies were reported in English. Of the 10 articles [17–19, 21–27], 1 described case-control design and 5 described cohort design involved Western region, and 4 described cohort design involved Asian region. Moreover, there were two publications focusing on SCLC, five on NSCLC, one on mixed cancers including SCLC and NSCLC, and two with unavailable information concerned. Of the 10 included publications, adjusted multivariate analyses for the effect of metformin use on the survival outcome of lung cancer patients with diabetes were performed in 7 publications, and unadjusted univariate analyses was performed in 3 publications. Totally, 6 individual studies reporting the relevant HRs with 95%CIs for the effect of metformin use on DFS and 11 individual studies concerning the effect of metformin use on OS were enrolled. Detailed descriptive data for studies included in our meta-analysis are presented in Table 1. According to the defined quality assessment, all studies were rated at six to eight triangles, which suggests a good quality(Table 1S).

**Table 1:** Characteristics of the studies included in the meta-analysis

First author, year	Country	Ethnicity	Study	Reference	Subtype	Stage	Sample size	Treatment	Adjusting variables	HR(95%CI)	HR(95%CI)
			design	group			N			for DFS	for OS
Ahmed,2015[19]	USA	Western	cohort	Nonmetformin	NSCLC	I-IV	40	Chemoradiation	NR	1.40 (0.65-3.04)	1.73 (0.78-3.85)
Kong,2015[21]	China	Asian	cohort	Nonmetformin	SCLC	Limited to Extensive	259	Chemotherapy	Performance status,stage	0.504 (0.286–0.889)	0.485 (0.314–0.750)
Cuurie,2012[22]	UK	Western	cohort	Nonmetformin	NR	NR	7345	NR	Age, sex, smoking history,	-	0.767 (0.590–0.997)
									Townsendindex of deprivation		
									Charlson comorbidity index		
									number of primary care contacts		
									year of diagnosis		
Xu,2015[18]	China	Asian	cohort	Nonmetformin	SCLC	Limited to Extensive	79	Chemotherapy	Performance status,stage	0.568(0.359–0.897)	0.549 (0.382–0.789)
Tan,2011[17]	China	Asian	cohort	Nonmetformin	NSCLC	II-IV	99	Chemotherapy	NR	0.762(0.641-0.905)	0.65(0.475-0.889)
Lin,2015[23]	USA	Western	cohort	Nonmetformin	NSCLC	IV	750	Comprehenxive	stage, cancer characteristics,	-	0.80 (0.71-0.89)
									chemotherapy use		
									tumor histology, tumor site,		
									receipt of PET scan		
Chen,2015[24]	China	Asian	cohort	Nonmetformin	NSCLC	III-IV	90	TKI	NR	0.46(0.28-0.75)	0.44(0.26-0.76)
Mazzone,2012[25]	USA	Western	cohort	Nonmetformin	Mixed	NR	507	NR	stage and age	-	1.47 (1.12-1.92)
Xu,2015[26]	USA(1)	Western	case-control	Nonmetformin	NR	NR	NR	NR	age, sex, race, BMI, tobacco use,	-	0.90(0.64-1.28)
	USA(2)	Western	case-control	Nonmetformin	NR	NR	NR	NR	insulin use, Charlson index	-	0.76(0.58-0.99)
Wink,2016[27]	Germany	Western	cohort	Nonmetformin	NSCLC	II-III	682	chemoradiotherapy	age,gender,performance status,stage	0.63(0.41-0.96)	0.86(0.57-1.28)

NSCLC, non-small cell lung cancer; SCLC, small cell lung cancer; DFS, disease-free survival; OS, overall survival; HR, hazard ratio; 95%CI, 95% confidence interval; NR, not reported; “Nonmetformin” group, patients treated with other hypoglycemic drugs but not metformin.

### Quantitative synthesis

**Figure 2:**
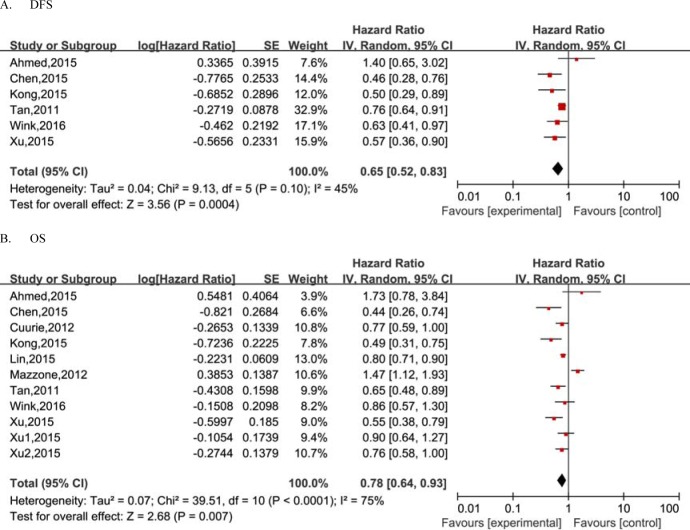
Meta-analysis of the effect of metformin use on survival outcomes of lung cancer patients with diabetes

**Figure 3:**
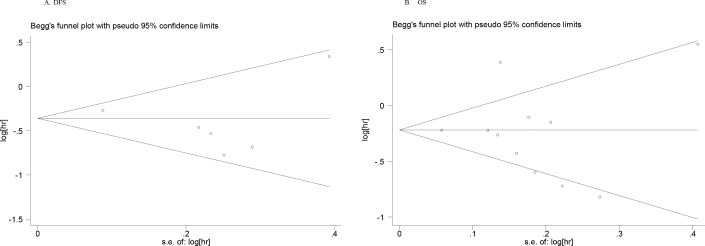
Begg's funnel plots for publication bias test on the association of metformin use with survival outcomes of lung cancer patients with diabetes

The pooled HR for association between DFS of lung cancer and exposure to metformin is shown in Table 2 and Figure 2. Overall, the results showed that metformin use was associated with a decreased risk of recurrence or progression in lung cancer patients with diabetes (HR = 0.65, 95%CI = 0.52-0.83) with the random effect model due to evident heterogeneity between studies. Considering the large variations in the study, we performed Begg's funnel plot (no obvious asymmetry observed in Figure 3) and Egger's test (*p* = 0.513), which suggested no evidence of publication bias. Stratification analyses based on region revealed a reduced risk of recurrence or progression in lung cancer patients with diabetes for metformin use in Asian region (HR = 0.69, 95%CI = 0.59-0.80) with a fixed effect model. However, no such significant association was observed in Western region. In the subgroup analysis by treatment strategy, the results showed metformin administration was significantly associated with decreased risk of recurrence or progression in patients receiving chemotherapy (HR = 0.71, 95%CI = 0.64-0.83), while surprisedly, no such significant survival benefit was reveled in patients receiving chemoradiotherapy. Furthermore, we also performed subgroup analyses by subtype of lung cancer. Our results demonstrated a significant association between metformin administration and decreased risk of recurrence or progression (for SCLC: HR = 0.54, 95%CI = 0.38-0.77; for NSCLC: HR = 0.70, 95%CI = 0.51-0.96).

**Table 2:** Meta-analysis results of the associations between metformin use and survival of lung cancer patients with diabetes

Survival	Subgroup		Number of		Pooled analyses	*P* value	heterogeneity		Effect
			studies		HR(95%CI)		P_h_	I^2^	model
DFS	Overall			6	0.65 [0.52, 0.83]	0.0004	0.1	45%	R
	Ethnicity	Asian	4		0.69 [0.59, 0.80]	<0.001	0.13	47%	F
		Western	2		0.88 [0.41, 1.90]	0.74	0.08	68%	R
	Treatment strategy	Chemoradiotherapy	2		0.88 [0.41, 1.90]	0.74	0.08	68%	R
		Chemotherapy	3		0.71 [0.61, 0.83]	<0.001	0.23	32%	F
	Subtype	NSCLC	4		0.70 [0.51, 0.96]	0.03	0.08	56%	R
		SCLC	2		0.54 [0.38, 0.77]	0.0007	0.75	0%	F
OS	Overall		11		0.78 [0.64, 0.93]	0.007	<0.001	75%	R
	Ethnicity	Asian	4		0.55 [0.46, 0.67]	<0.001	0.55	0%	F
		Western	7		0.93 [0.76, 1.13]	0.45	0.002	71%	R
	Treatment strategy	Chemoradiotherapy	2		1.00 [0.69, 1.44]	0.98	0.13	57%	F
		Chemotherapy	3		0.58 [0.47, 0.71]	<0.001	54	0%	F
		Other	6		0.84 [0.66, 1.06]	0.13	<0.001	79%	R
	Subtype	NSCLC	5		0.75 [0.58, 0.97]	0.03	0.04	60%	R
		SCLC	2		0.52 [0.39, 0.69]	<0.001	0.67	0%	F
		Other	4		0.94 [0.68, 1.29]	0.69	0.002	80%	R

NSCLC, non-small cell lung cancer; SCLC, small cell lung cancer; DFS, disease-free survival; OS, overall survival; HR, hazard ratio;

95%CI, 95% confidence interval; *P*h, *p* value of the Q test for heterogeneity. R, random effect model; F, fixed effect model.

Considering the effect of metformin administration on the OS, as shown in Table 2 and Figure 2, a total of 11 individual studies were pooled into the meta-analysis. The overall estimate showed a significantly reduced risk of death for metformin administration (HR = 0.78, 95%CI = 0.64-0.93). Similarly, as shown in Figure 3, the Begg's funnel plot for publication bias detection showed no evident asymmetry, indicating the absence of publication bias, which was also supported by the Egger's test (*p* = 0.763). Analyses stratified by treatment strategy showed a reduction in the risk of cancer-related mortality in patients receiving chemotherapy(HR = 0.58, 95%CI = 0.47-0.71), while no reduced risk was presented in patients receiving chemoradiotherapy. The stratification analyses by region showed that metformin was associated with reduced death risk in Asian region (HR = 0.55, 95%CI = 0.46-0.67) but not in Western region. In the subgroup analyses based on subtype of lung cancer, the relative survival benefit associated with metformin remained in both SCLC and NSCLC subtype (HR = 0.52, 95%CI = 0.39-0.69; HR = 0.75, 95%CI = 0.58-0.97, respectively).

### Sensitivity analyses

Considering the large variations across studies, we performed sensitivity analysis by sequential omission of individual studies to recalculate the pooled HR for the remainder of the studies, and none of the exclusions of a specific study would substantially alter the trend of our primary pooled results, showing a significant association of metformin administration and improved DFS. Also, the pooled result was not materially affected in the sensitivity analysis concerning the OS.

## DISCUSSION

Our meta-analysis pooling currently available observational studies was designed to comprehensively and quantitatively evaluate the relationship between metformin use and the clinical outcome of lung cancer patients with diabetes, demonstrating a significant association of metformin therapy with improved DFS and OS.

Increasing evidence has strengthened the effects of metformin on prevention and treatment of lung cancer in recent years though the exact molecular mechanism for the anti-cancer role of metformin has not been fully unveiled to date. In vitro studies found that metformin inhibited the proliferation and cell cycle, and induced apoptosis in different lung cancer cells [28]. The nude mice study also provided further evidence indicating that the combined treatment of metformin with the mostly common agent cisplatin for lung cancer was capable of enhancing the anti-tumor efficacy [12]. Proposed potential molecular mechanisms have been suggested for the anti-neoplastic action of metformin by some experimental data [29–32]. There was a extensively accepted mechanism of metformin action that metformin could activate the LKB1/AMPK signal axis in lung cancer cell, subsequently resulting in reduced cancer cell growth and proliferation via the suppression of mTOR activity [15]. Moreover, it was also reported that metformin was able to depress IL-6-induced EMT possibly by blocking STAT3 phosphorylation, thereby inhibiting the growth and metastasis of lung adenocarcinoma [14]. Additionally, metformin was found to possibly underlie its ability to prevent the development of lung cancer by reducing circulating levels of IGF-I and insulin and mediate its effects through inhibition of the IGF-I/insulin receptor signaling and receptor tyrosine kinases (RTK) signal pathways [33, 34]. Over the recent years, a series of observational investigations has reported that metformin might modulate the clinical outcomes of lung cancer patients with diabetes, however obtained inconsistent or even controversial results probably due to the sample size, study design or other variation affecting the prognosis, which prompted us to perform comprehensive analyses to address the issues concerned.

In the present meta-analysis, metformin treatment was demonstrated to prolong the DFS and OS of lung cancer patients with diabetes, which was consistent with the previous meta-analysis [20]. In stratified analyses by subtype of lung cancer, metformin use was found to be significantly associated with a better prognosis both in SCLC and in NSCLC. Moreover, stratification by region showed that such survival benefit was only observed in Asian country, indicating that ethnicity variation may contribute to the effects of metformin on the survival of lung cancer, however, we are difficult to clarify the association in other non-Asian populations from western countries since a mixed population was enrolled in most original studies. Furthermore, the subgroup analyses based on treatment strategy revealed a more favorable prognosis in patients receiving chemotherapy, but this beneficial effect did not appear in patients receiving chemoradiotherapy, which may be due to the less studies(only 2 studies) and sample size included in chemoradiotherapy subgroup, indicating the necessity to perform more investigations regarding the issue based on different treatment strategies. Collectively, the findings in overall analyses and subgroup analyses of the current study regarding the association of metformin use with OS of lung cancer patients with diabetes were in line with the previous meta-analysis [20]. Nevertheless, the present meta-analysis was, comparatively, considered more convincible and reliable with several advantages than the previous [20]. First, more individual studies regarding the association of metformin use with OS of lung cancer patients with diabetes were enrolled in the study, which would reduce the bias and strengthen the conclusions. Second, we have concluded for the first time the association of metformin use with DFS of lung cancer patients with diabetes with a relatively large sample size, which was not investigated in the previous meta-analysis [20]. Third, we have performed subgroup analysis based on treatment strategy, providing new findings for this association. In addition, no publication bias was indicated and the results of sensitivity analysis were also relatively stable, which both suggested the robustness of the results in the present study.

In spite of the advantages, caution is called for the appropriate interpretation of our findings with the consideration of several potential limitations existing in the study. First, although adjusted estimates were largely utilized in the meta-analysis, the adjusted confounders varied across studies and some potential residual or unknown confounders might not be fully adjusted, which may affect the survival of cancer patients and thus bias the effect estimate of metformin on survival. We also used unadjusted estimates for the study to minimize potential bias because we failed to obtain the individualized primary data from the included studies, though we have tried to contact the authors for data request. Second, details including metformin dose, and other significant information relevant to cancer prognosis, especially including tobacco use [35], beside subtype, cancer stage and exhaustive treatments protocols for cancer patients in some studies were incomplete, potentially resulting in the observed heterogeneity between studies and making the subgroup analyses less effective and the results therefore should be interpreted with cautions. Third, the diversity in study populations, particularly in western country may translate into a substantial heterogeneity across studies in effect estimates. Fourth, it may be too simple to classify patients according to metformin exposure versus nonmetformin-exposure. Most patients with diabetes are treated with one or more hypoglycemic agents with changes in pharmacotherapy and severity of diabetes over time [36]. The possible dose-response relationship and time-related effect involved in the observed survival benefit in metformin users is difficultly determined by the simple comparators, which, however, was usually explored by few studies. Fifth, Asian population is retrieved only from China according to the inclusion criteria, which would possibly limit the representativeness of conclusions, suggesting the necessity to verify and replicate the results in other Asian countries. Therefore, the association between metformin treatment and a favorable prognosis may be affected to some extent.

In summary, our meta-analysis demonstrated a significant association of metformin use with a favorable survival outcome, suggesting a potentially available choice of treatments for lung cancer patients with diabetes. Meanwhile, the current study reveals the need for more blind randomized controlled clinical trials to further assess the efficacy of an antidiabetic regimen, metformin in the treatment of lung cancer.

## MATERIALS AND METHODS

### Search strategy

We searched PubMed, Embase, Web of Science, Cochrane Library, Wan Fang, China National Knowledge Infrastructure, and the Chinese BioMedical Literature databases for relevant studies investigating the association between metformin use and the survival outcome of lung cancer patients with diabetes up to March 2016 without language restrictions. We developed a search strategy using the following terms in various combinations: [“metformin” OR “biguanides”] and [“lung cancer” OR “lung carcinoma”]. We also screened the reference lists of identified studies and reviews for additional relevant studies.

### Eligibility criteria

Studies were considered eligible if they fulfilled the following criteria: (1)case-control study or cohort study assessing the association of metformin use and prognosis of lung cancer patients with diabetes. (2) studies contained time to event data (hazard ratios [HRs] with 95% confidence interval [CI]) pertaining to association between metformin use and survival of lung cancer patients with diabetes. Studies with a small sample size and low study quality or no time to event data provided were excluded. In case of duplication, only the study with the most complete information and largest sample size was selected.

### Data extraction and quality assessment

For each included study, the following information was independently extracted by two investigators: first author, year of publication, region, country, study design, reference of group, subtype of lung cancer, clinical stage, sample size, treatment choice of patients, adjusting variables, HRs with their 95 % CIs for DFS and OS. For observational studies, the results from both unadjusted and adjusted models (adjusted for the largest number of potential confounders) were likely selected. If several estimates were reported in the same article, we select the most fully adjusted estimate (i.e., multivariate analysis was selected over univariate analysis). If an article reported multiple estimates by subgroup only, these estimates were entered separately into our relevant meta-analysis data set. Any disagreement was resolved by discussion or consultation with a third reviewer to reach a consensus. The Newcastle-Ottawa quality assessment scale was applied in the quality assessment of included studies, concerning some of important factors described as the previous studies [37, 38].

### Statistical analysis

Pooled HRs and associated 95%CIs were calculated using a fixed or random effect model according to the heterogeneity across the studies with Review Manager version 5.2 software (provided by The Cochrane Collaboration, Oxford, UK; http://www.cochrane.org/software/revman.htm). The significance of the pooled HR was determined by Z test and *p* < 0.05 was considered significant. Inter-study heterogeneity across studies was assessed using the Cochran's χ^2^-based Q test and the *I*-squared test [39], and p>0.10 or I^2^ < 50% indicated the absence of heterogeneity, then the fixed effect model was selected to calculate the pooled HRs [40], otherwise, the random effect model was used [41]. The subgroup analyses by the potentially important compounding factors such as region, subtype of lung cancer and treatment strategy, were also performed to explore the source of possible heterogeneity. According to the treatment of reference group, we defined the participants with other hypoglycemic drugs but not metformin as “Nometformin” group, the participants with metformin with or without other agents as “Metformin” group. Potential publication bias was assessed by visually examining the Begg's funnel plot asymmetry, which was further assessed by Egger's linear regression test (*p* < 0.05 indicated the presence of publication bias) [42, 43], using Stata 12.0 software (Stata Corp., College Station, USA). We also conducted the sensitivity analysis by sequential omission of individual studies to assess the stability of results.

## SUPPLEMENTARY MATERIAL TABLE


