# Supplementary material for: Metformin therapy associated with survival benefit in lung cancer patients with diabetes
Source: Oncotarget. 2016 Apr 20;7(23):35437–45. doi: 10.18632/oncotarget.8881 (PMC5085241; doi:10.18632/oncotarget.8881)
Supplement: Supplementary file 1 [file oncotarget-07-35437-s001.pdf]

# Metformin therapy associated with survival benefit in lung cancer patients with diabetes

**Supplementary Material**

Table 1S. The quality assessment of the included studies appraised in reference to the Newcastle-Ottawa statement

| First author, year | Country | Selection (4) | | | | Comparability (2) | | Outcome (3) | | | Total |
|---|---|---|---|---|---|---|---|---|---|---|---|
| Ahmed,2015[19] | USA | ▲ | ▲ | ▲ | | ▲ | ▲ | ▲ | ▲ | ▲ | 8 |
| Kong,2015[21] | China | ▲ | ▲ | | | ▲ | ▲ | ▲ | ▲ | ▲ | 7 |
| Cuurie,2012[22] | UK | ▲ | ▲ | | | ▲ | ▲ | ▲ | ▲ | | 6 |
| Xu,2015[18] | China | ▲ | ▲ | | | ▲ | ▲ | ▲ | ▲ | ▲ | 7 |
| Tan,2011[17] | China | ▲ | ▲ | | | ▲ | ▲ | ▲ | ▲ | | 6 |
| Lin,2015[23] | USA | ▲ | ▲ | ▲ | | ▲ | ▲ | ▲ | ▲ | ▲ | 8 |
| Chen,2015[24] | China | ▲ | ▲ | ▲ | | ▲ | ▲ | ▲ | ▲ | | 7 |
| Mazzone,2012[25] | USA | ▲ | ▲ | ▲ | ▲ | ▲ | | ▲ | ▲ | | 7 |
| Xu,2015[26] | USA | ▲ | ▲ | ▲ | | ▲ | | ▲ | ▲ | | 6 |
| Wink,2016[27] | Germany | ▲ | ▲ | ▲ | | ▲ | ▲ | ▲ | ▲ | | 7 |
